# Application of Single-Cell Sequencing and Machine Learning in Prognosis and Immune Profiling of Lung Adenocarcinoma: Exploring Disease Mechanisms and Treatment Strategies Based on Circadian Rhythm Gene Signatures

**DOI:** 10.3390/cancers17172911

**Published:** 2025-09-05

**Authors:** Qiuqiao Mu, Han Zhang, Kai Wang, Lin Tan, Xin Li, Daqiang Sun

**Affiliations:** 1 Clinical School of Thoracic, Tianjin Medical University, Tianjin 300070, China; mqqfighting@163.com; 2 Department of Thoracic Surgery, Tianjin Chest Hospital, Tianjin 300222, China; 3 Qingdao Hospital, University of Health and Rehabilitation Sciences, Qingdao 266113, China

**Keywords:** cancer immunotherapy, tumor microenvironment, gene therapy, LUAD, circadian rhythm-related genes

## Abstract

**Background**: The circadian rhythm regulates important functions in the body, such as metabolism, the cell cycle, DNA repair, and immune balance. Disruption of this rhythm can contribute to the development of cancer. Circadian rhythm genes (CRGs) are attracting attention for their connection to various cancers. However, their roles in LUAD are not yet well understood. Additionally, our knowledge of how they function at both the bulk tissue and single-cell levels is limited. This gap hinders a complete understanding of how CRGs impact the development and outcomes of LUAD. **Methods**: We selected 554 CRGs from public databases. We then obtained transcriptome data from TCGA and GEO. A total of 101 machine learning algorithm combinations were tested using 10 algorithms and 10-fold cross-validation. The best-performing model was based on Stepwise Cox regression and SuperPC. This model was validated with additional datasets. We also examined the relationships between CRGs, immune features, tumor mutation burden (TMB), and the response to immunotherapy. Drug sensitivity was also assessed. Single-cell data identified the cell types with active CRGs. Next, we performed qRT-PCR and other basic experiments to validate the expression of ARNTL2 in LUAD tissues and cell lines. The results indicated that ARNTL2 may play a key role in lung adenocarcinoma. **Results**: The CRG-based model clearly distinguished LUAD patients based on their risk. High-risk patients exhibited low immune activity, high TMB, and poor predicted responses to immunotherapy. Single-cell data revealed strong CRG signals in epithelial and fibroblast cells. These cell groups also displayed different communication patterns. Laboratory experiments showed that ARNTL2 was highly expressed in LUAD. It promoted cell growth, movement, and invasion. This suggests that ARNTL2 may play a role in promoting cancer. **Conclusions**: This study developed a machine learning model based on CRGs. It can predict survival and immune status in LUAD patients. The research also identified ARNTL2 as a key gene that may contribute to cancer progression. These findings highlight the significance of the circadian rhythm in LUAD and provide new perspectives for diagnosis and treatment.

## Simple Summary

This study explores the role of circadian rhythm-related genes (CRGs) in lung adenocarcinoma (LUAD) and their potential impact on tumor immunity. By integrating advanced machine learning techniques with bulk and single-cell transcriptomic data, we developed a robust CRG-based prognostic model. Specifically, we evaluated 101 machine learning models built using 10 algorithms and 10-fold cross-validation, and ultimately constructed a stable signature through stepwise Cox regression and Supervised Principal Components (SuperPC). The model’s performance was validated across multiple independent cohorts, consistently outperforming conventional clinical markers and previously published models, suggesting its promising translational potential. To further dissect the immunological landscape, we incorporated bulk RNA-seq and single-cell RNA-seq analyses. Our findings revealed that patients stratified into the high-risk CRG group exhibited reduced immune activity, elevated tumor mutation burden (TMB), and poorer predicted response to immunotherapy (IPS and TIDE scores). Single-cell analysis identified pronounced CRG signals in epithelial and fibroblast populations, accompanied by distinct cell-cell communication patterns. Functional assays demonstrated that ARNTL2 is markedly upregulated in LUAD and promotes tumor proliferation, migration, and invasion, highlighting its potential as a cancer driver gene. Together, our study provides a novel circadian rhythm-related signature for prognostic stratification and immune phenotype prediction in LUAD, offering mechanistic insights into CRG-mediated tumor-immune interactions and potential avenues for integrating circadian biology into cancer immunotherapy.

## 1. Introduction

LUAD is the most frequent subtype of non-small cell lung cancer and continues to pose a significant global health burden because of its high occurrence and poor prognosis [1,2,3,4]. Although treatments like immunotherapy and targeted therapy have improved, the prognosis for LUAD patients is still poor. This is mainly because the disease shows high molecular diversity and complex biology. The tumor microenvironment typically exhibits a range of genetic, epigenetic, and cellular alterations, making the disease challenging to decipher, diagnose, and manage [5,6,7]. As a result, there is an urgent need to find dependable molecular markers. These markers can help guide personalized treatment plans and make risk grouping more accurate.

The circadian rhythm is an internal timing system that controls many body functions like hormone signals, metabolism, DNA repair, cell cycle, and immune balance [8,9,10]. CRGs play a key role in keeping gene activity in sync across different tissues. When CRGs are disrupted, they may lead to cancer by changing how cells grow, die, use energy, and respond to the immune system [11]. But there is still no full study on CRGs in LUAD that includes both multi-cohort validation and single-cell analysis [12,13].

Although LASSO combined with Cox regression is a common method for building prognostic models, it has several limits. It is simple, easy to overfit, sensitive to noise, and not good at generalizing across datasets. To fix these problems, we used a machine learning framework that brings together multiple algorithms. We ranked the models based on the C-index. This helped us find the best model with higher accuracy and better stability across different datasets.

ScRNA-seq provides a detailed approach to explore tumor composition. It helps reveal the diverse cell types and their functions within the tumor environment. It helps researchers see different cell types, their gene activity, and how they interact in the tumor environment. This method shows details that bulk RNA data cannot provide and helps find new treatment targets and disease mechanisms [14,15,16,17]. In this study, we built a gene signature linked to circadian rhythm using machine learning. We tested it in different LUAD datasets to make sure it works well and is reliable. To learn more about how this signature works, we used scRNA-seq to look at CRGs at the single-cell level. We also did qRT-PCR on LUAD samples from Tianjin Chest Hospital to check the model genes. After knocking down these genes in A549 and H1299 cells, we found that they may help cancer growth. These results support the importance of the model genes.

In summary, this study provides a clear and comprehensive view of how genes related to circadian rhythm function in LUAD. It explains how these genes may influence the development and progression of the disease. We built a stable and repeatable scoring model to show the value of CRGs in predicting outcomes. This model also shows that CRGs are closely linked to tumor features like immune cells, mutation load, and treatment response. We confirmed these findings using both bulk and single-cell RNA data. These results give new ideas and suggest possible gene targets for better ways to prevent, diagnose, and treat LUAD based on circadian rhythm.

## 2. Method

### 2.1. Strategy for Transcriptomic Data Acquisition and Correction of Batch Effects

The scRNA-seq data used in this study came from a published and publicly available dataset [18]. This dataset included paired samples of primary tumor tissues and nearby non-cancerous lung tissues from ten patients with LUAD. These matched samples made it possible to clearly compare the cell types and gene expression patterns between cancer and normal tissues. The raw sequencing files were downloaded from the data repository provided in the original publication. For bulk RNA-seq analysis, we collected transcriptome data of LUAD patients from The Cancer Genome Atlas (TCGA) using the TCGAbiolinks R package, which included gene expression levels, clinical information, and somatic mutation data [19]. In addition to the main dataset used for building the model, we also retrieved six independent transcriptome datasets from the GEO database. These datasets were used as validation cohorts. They came from different research platforms and patient groups. We used them to test how well the model performed in different clinical and technical settings. All datasets used in this study are detailed in Supplementary Table S1. To ensure consistency of expression levels across samples, raw count data were normalized and converted into TPM format. Batch effects across different datasets were corrected using the ComBat method implemented in R (sva package, version: 3.57.0) [20,21,22]. This method helped make the data more comparable across studies.

### 2.2. Identification of a Circadian Rhythm-Driven Gene Signature for Prognostic Stratification

To identify genes related to circadian rhythm regulation, we first collected a curated list of circadian genes from the Molecular Signatures Database (MSigDB). We selected gene sets that were labeled with terms like “circadian”, “circadian rhythm”, or “biological clock”. This method ensured that all included genes had well-documented roles in circadian processes and provided a biologically meaningful base for the following analysis [23,24]. Then we extracted the gene symbols for further study. We analyzed the TCGA-LUAD dataset and found genes with significant expression changes from the circadian-related gene set. These genes showed clear differences between tumor and normal samples. Next, we applied univariate Cox analysis to identify genes associated with patient survival. This step was used to assess their potential as prognostic indicators. To build a stable prognostic model, we followed a machine learning framework proposed by Liu et al. [25]. This framework included ten survival prediction methods: Lasso, Ridge, Elastic-net (Enet), CoxBoost, Stepwise Cox, plsRcox, SuperPC, Survival-SVM, Random Survival Forest (RSF), and Gradient Boosting Machine (GBM). We combined these methods in various ways to create 101 different machine learning algorithm combinations. Each model included a step for feature selection and a step for survival prediction. We evaluated the model’s performance using the C-index. In contrast to the LOOCV method adopted by Liu and colleagues, our study employed a 10-fold cross-validation approach. We randomly divided the dataset into ten equal parts. Each time, we trained the model on nine parts and tested it on the remaining one. We repeated this process ten times. This approach helped improve statistical reliability and reduced the chance of overfitting. It also gave a better estimate of how well the model could generalize. We identified the StepCox combined with SuperPC as the optimal model by averaging C-index values across external validation cohorts. To stratify patients, we used the median value of the calculated risk score and divided them into two categories: high-risk and low-risk. Survival differences between the two groups were evaluated using Kaplan–Meier plots and receiver operating characteristic (ROC) analysis. In addition, we benchmarked our model against 114 previously reported LUAD signatures to assess its predictive performance.

### 2.3. External Validation Datasets

To evaluate the generalizability and robustness of our CRGs-based model, we used multiple external validation datasets. These datasets were selected because they are relevant to the model’s features and are consistent with the training dataset, allowing us to test the model’s performance across different patient populations and clinical settings.

The external datasets used in this study include publicly available transcriptomic data from LUAD patients, such as GSE13213, GSE26939, GSE30219, GSE31210, GSE42127, and GSE50081, among others. These datasets provide key clinical information, including survival data. They offer an opportunity to evaluate the CRGs model in independent cohorts, providing validation beyond the training data.

The selected datasets were carefully chosen based on their similarity to the training and testing datasets, particularly in terms of features such as gene expression profiles, clinical variables, and patient demographics. By including external cohorts with comparable features, we ensure that the model’s predictive accuracy is not dependent on a single dataset, enhancing its applicability and robustness.

Validating the model across these diverse datasets has further strengthened its ability to predict survival outcomes and provided a more comprehensive assessment of its performance in different clinical contexts.

### 2.4. Clinical Correlation and Prognostic Evaluation

In the TCGA cohort, we divided patients into high-risk and low-risk groups based on the median value of the risk score calculated from our prognostic model. This grouping helped us compare survival outcomes between the two groups. To show how the model works in a clinical setting, we made clear visual plots. These plots showed the individual risk scores assigned to each patient. They also showed the survival outcome and the expression levels of model-related genes. These visualizations provided a simple way to understand how well the model separates patients and what biological patterns it may reflect. To examine the relationship between survival and clinical or molecular features, we applied both univariate and multivariate Cox regression analyses. We used forest plots to display these outcomes. The plots were first generated with base R, then refined using the ggplot2 package for better readability. In addition, we created a nomogram that combined tumor stage and risk score to estimate survival probability for individual patients. This tool estimates the chances of survival at 1, 3, and 5 years. We tested its accuracy by using 1000 bootstrap samples for each time point. We also used decision curve analysis (DCA) to check how useful the nomogram is in practice. We compared its net benefit with using single predictors alone. Finally, we drew calibration plots to compare predicted survival with real outcomes at 1, 2, and 3 years. This helped confirm that the model gives consistent and reliable results over time.

### 2.5. Analysis of Immune Cell Infiltration and the Tumor Microenvironment

To evaluate the immune infiltration landscape, we used several computational tools. These included TIMER, CIBERSORT, CIBERSORT-ABS, QUANTISEQ, MCPcounter, XCELL, and EPIC. These tools extracted immune cell infiltration scores from normalized gene expression data and matched them with the risk groups [26,27,28,29,30,31]. Before visualization, we applied z-score transformation to the expression data. We also limited the values within ±2 standard deviations. Then we used the ComplexHeatmap R package to create a heatmap that showed immune infiltration patterns and risk classification [32]. To assess pathway activity differences between samples, we performed gene set variation analysis with the GSVA R package. GSVA converted gene-level expression into pathway-level scores. This helped us better understand functional differences between sample groups. We used the TCGA-LUAD cohort’s TPM-normalized expression data to perform ssGSEA. This allowed us to assess the activation level of immune-related pathways in each individual sample [33]. We applied the limma package in R to compare pathway activities between the high-risk and low-risk groups. This analysis helped us identify which pathways showed clear differences between the two groups. This method fit linear models to the GSVA scores and gave stable comparison results. We considered pathways with an absolute t-value greater than 3 (|t-value| > 3) to be statistically significant. We used ranked bar plots to visualize these results and highlight the most changed pathways between the two groups [34]. We used the ESTIMATE method to assess features of the tumor microenvironment. This approach generated values for stromal content, immune cell infiltration, and tumor purity in each sample. Then, we examined the association between these indicators and the CRGs risk score [35]. These results helped us understand the strength and direction of each relationship. We used the ggplot2 package to make scatter plots and correlation heatmaps. These plots helped make the results clearer and easier to understand.

### 2.6. Single-Cell Atlas Construction and CRGs Activity Scoring

We processed the scRNA-seq data using the Seurat workflow [36]. First, we read the raw expression matrices in 10× Genomics format and merged them across samples. Then we calculated the percentages of mitochondrial genes and hemoglobin genes. We filtered cells based on the following criteria: gene count between 500 and 10,000, UMI count between 1000 and 100,000, mitochondrial content below 40%, and hemoglobin content below 5%. We used violin plots to show the quality control results. Next, we used NormalizeData and FindVariableFeatures to normalize the data and find highly variable genes. We calculated cell cycle scores using classic S phase and G2M phase marker genes. We also removed the effect of cell cycle during normalization. We performed PCA for dimensionality reduction to show the global transcriptional pattern and identify major sources of variation across samples. To correct for batch effects caused by technical or experimental differences, we used the Harmony algorithm. This method aligned shared biological signals and reduced unwanted variation. It improved the consistency and interpretability of later analyses. We used the top 20 Harmony components for clustering. We applied the Louvain algorithm for clustering and used UMAP for visualization. We annotated cell identities based on classic marker genes and confirmed them with dot plots. We curated final cell type labels based on marker expression and saved them in the metadata. We calculated the proportion of each cell type in each sample and displayed it using stacked area plots. We scored the CRGs at the single-cell level using five methods: AUCell, UCell, singscore, ssGSEA, and AddModuleScore [37]. We extracted both raw and normalized expression values. We scaled all scores to a 0–1 range. We defined a combined score as the sum of all scaled values. We showed the overall CRGs activity using dot plots, UMAP projection, and violin plots across cell types. For the next steps in analysis, we grouped the cells into two categories: CRGs_high and CRGs_low. The grouping was based on the median value of the CRGs score, which served as the cutoff point. This grouping helped us compare transcriptional features and functions between cells with high or low CRG activity. It also helped us explore the biological heterogeneity linked to circadian rhythm at the single-cell level.

### 2.7. Intercellular Communication Profiling

We applied the CellChat tool to examine how various cell types interact with each other. This method helped us analyze the communication signals exchanged among different cell groups [38]. We divided the single-cell dataset into two groups based on CRG scores: high and low. The classification was based on the median circadian rhythm gene score. This approach allowed us to compare cells with different CRG activity levels. We analyzed each group separately. For each group, we combined the expression data with the curated cell type annotations into a CellChat object. We used the human ligand-receptor interaction database (CellChatDB.human) to build the signaling network [39]. We started by identifying highly expressed ligands and receptors. Then we inferred significant interactions and mapped them onto the protein–protein interaction (PPI) network. We used permutation testing to calculate the probability of each interaction. We also scored and summarized all signaling pathways based on network centrality. We first combined the CellChat results from the two risk groups. After that, we analyzed and compared how many cell interactions were predicted and how strong those interactions were. This helped us understand the communication differences between the groups. We used heatmaps and circular network plots to show the differences in interaction patterns. We also checked changes in sending and receiving signals. We used signaling role scatter plots and signalingRank comparison to display these changes. For specific pathways, we used bubble plots and signaling role network maps. We used violin plots to show how key signaling genes were expressed in each group. This allowed us to clearly compare the expression patterns between the two risk groups. We compared the expression levels of key signaling genes between groups using violin plots.

### 2.8. Integration of Copy Number Variation and Mutation Burden for Prognostic Stratification

We used the output files from GISTIC2.0 to evaluate somatic copy number alterations (SCNAs) and calculated G-scores to identify significant amplification and deletion events across the genome [40]. We analyzed mutation features based on binary mutation matrices, tumor mutational burden (TMB), mutational signatures, and copy number variation data. We selected the most frequently mutated genes, along with arm-level and gene-level CNAs, to describe group-specific genomic changes. We log-transformed TMB values before comparing them between groups. We examined how the risk score was related to TMB by calculating their correlation. After that, we used the best cutoff point to separate patients into two groups: TMB-high and TMB-low. Finally, we performed joint survival analysis based on both TMB status and risk classification.

### 2.9. Multidimensional Analysis of Predictive Features for Immunotherapy Response

To explore how different risk levels might respond to immunotherapy, we analyzed various immune-related characteristics. As a first step, we examined the expression patterns of key immune checkpoint genes. Several immunosuppressive markers were found to be more highly expressed in patients with high-risk scores. We then tested correlations between these genes and the risk score. Many of them showed a positive correlation, which suggests they may work together to build an immunosuppressive tumor environment. Next, we evaluated tumor immunogenicity using Immunophenoscore (IPS) data from the TCIA platform [41]. The immune microenvironment in low-risk patients appeared more active and responsive. This was reflected by higher levels of cytolytic activity and increased expression of MHC-related genes. These observations indicate that low-risk individuals may mount a stronger immune defense against tumors, which may lead to better clinical outcomes. Similar findings were seen in the TIDE analysis. High-risk patients showed elevated exclusion scores, suggesting greater immune evasion and reduced infiltration of lymphocytes. Taken together, these results suggest that high-risk patients may have an immune-resistant phenotype. This could limit the success of immune checkpoint blockade therapies. The observed resistance might be caused by a suppressive immune environment or limited immune activation. In contrast, individuals classified in the low-risk group exhibited higher levels of immune response compared to others. Their immune environment was also more supportive of anti-tumor responses. This may help improve their sensitivity to immunotherapy. These differences highlight the value of risk stratification in guiding personalized immunotherapy strategies.

### 2.10. Assessment of Drug Response Variability Across Risk Subgroups

To explore differences in drug sensitivity between risk groups, we combined transcriptomic and pharmacogenomic data. We used the oncoPredict framework for the analysis. We obtained the data from the GDSC2 database. After performing initial processing and removing low-quality samples, we applied the calcPhenotype module to estimate drug sensitivity for each individual sample. Then, we analyzed the predicted scores by comparing the high-risk group with the low-risk group. To test if the differences in predicted responses were statistically significant, we used the Wilcoxon rank-sum test. This non-parametric test is suitable for comparing two independent groups with non-normal data. This method helped us identify drugs that may have different effects in the two risk groups. We visualized the drugs with significant response differences and marked them as potential candidates for personalized treatment.

### 2.11. Cultivation and Transfection of LUAD Cell Lines

The A549 and H1299 cell lines were originally derived from human lung adenocarcinoma. When cell density reached about 60% to 70%, transfection was carried out. Small interfering RNA (siRNA) was introduced using Lipofectamine 3000 (Invitrogen, Carlsbad, CA, USA), following the instructions provided by the manufacturer. After transfection, the cells were incubated under standard conditions for another 24 to 48 h. This step allowed enough time for gene silencing and helped reduce off-target effects before performing follow-up experiments. The siRNA oligonucleotides were synthesized by a commercial provider. The sequences are listed in Supplementary Table S2.

### 2.12. Quantitative Real-Time PCR

Fresh tumor samples were collected from lung adenocarcinoma tissues surgically removed at the Department of Thoracic Surgery, Tianjin Chest Hospital. All patients gave informed consent, and the study was approved by the ethics committee. Total RNA was extracted using TRIzol reagent. Reverse transcription was performed to make cDNA templates. Quantitative PCR was performed using SYBR Green dye on the ABI QuantStudio platform. Each experiment was repeated three times for every sample. GAPDH served as the reference gene. It was used to adjust and compare gene expression levels among different samples. The relative mRNA levels of target genes were calculated using the 2^−ΔCt^ method. Here, ΔCt is the difference between the Ct values of the target gene and the reference gene. This normalization helped reduce variation between samples and ensured the accurate measurement of gene expression.

### 2.13. Transwell Migration and Invasion Assays

Cell migration ability was tested using transwell inserts with 8.0 µm pores (Corning, Glendale, AZ, USA). In the migration assay, cells were suspended in serum-free RPMI-1640 medium and placed in the upper chamber. The lower chamber was filled with medium containing 10% fetal bovine serum to act as a chemoattractant. For the invasion assay, the upper side of the membrane was coated with Matrigel (BD Biosciences, Franklin Lakes, NJ, USA) to mimic the extracellular matrix. Cells were incubated for 24 to 48 h. After that, non-migrated or non-invaded cells were carefully removed using a cotton swab. After incubation, cells that passed through the membrane were fixed and stained with crystal violet solution (Solarbio, Beijing, China). Cells attached to the underside of the membrane—representing migratory or invasive populations—were then visualized and quantified under a microscope to assess their movement and invasion capacity.

### 2.14. Colony Formation Assay

LUAD cell lines A549 and H1299 were seeded at low density (500–1000 cells per well) into 6-well plates. The cells were grown in RPMI-1640 medium with 10% fetal bovine serum and antibiotics. The plates were kept under standard culture conditions (37 °C, 5% CO_2_) for 10 to 14 days. When visible colonies with more than 50 cells appeared, the cells were fixed with 4% paraformaldehyde for 15 min. Then the colonies were stained with 0.1% crystal violet for 30 min. The number of colonies was counted under a bright-field microscope.

### 2.15. Statistical Methods and Data Interpretation

We used the R programming language (version 4.2.1) to conduct bioinformatics-related analyses. For experimental data, visualization and statistical analysis were performed using GraphPad Prism version 9.0 and ImageJ version 1.53k. Group differences were tested using either Student’s *t*-test or the Wilcoxon rank-sum test, depending on the normality of the data. The Kruskal–Wallis test was applied when comparing three or more groups. It is a non-parametric approach that works well for evaluating distribution differences among multiple independent groups. To assess survival outcomes, we generated Kaplan–Meier survival curves to show survival probabilities over time. We used the log-rank test to assess whether the survival differences between groups were statistically meaningful. To explore the relationship between variables and survival, we applied the Cox regression model. Both univariate and multivariate analyses were performed. Spearman correlation was used to examine the associations between continuous variables. A *p*-value less than 0.05 (two-sided) was considered to show statistical significance.

## 3. Results

### 3.1. Genome-Wide Profiling of Circadian Genes Reveals Dysregulation and Survival Association in LUAD

**Figure 1:**
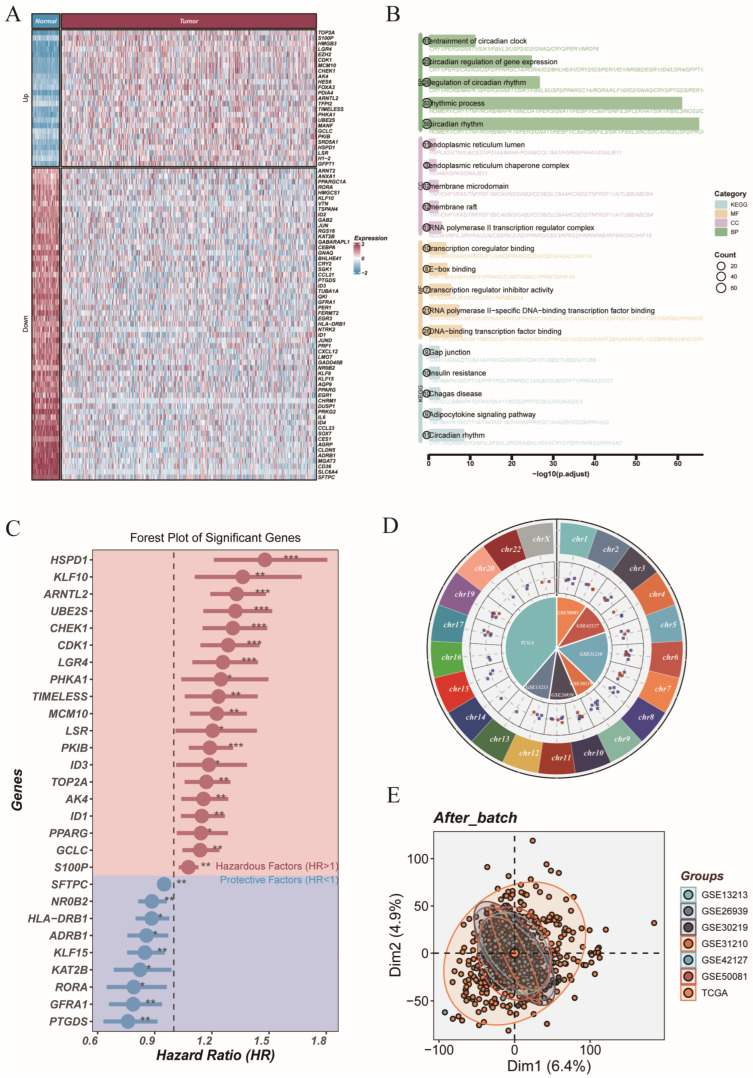
Multi-omics characterization of differentially expressed circadian rhythm-related genes in LUAD. (**A**) Heatmap showing the differential expression of circadian genes between tumor and normal samples in TCGA-LUAD cohort. (**B**) Functional enrichment analysis (GO and KEGG) of differentially expressed circadian rhythm-related genes. (**C**) Forest plot of significant genes identified by univariate Cox regression analysis; genes with HR > 1 are hazardous factors (red) and HR < 1 are protective factors (blue). (**D**) Chromosomal distribution of prognostic circadian genes. Outer rings show chromosomal locations, with red and blue indicating upregulated and downregulated genes in tumors, respectively; the inner circle denotes the gene presence across datasets. (**E**) PCA plot showing batch effect correction among TCGA and GEO datasets after harmonization. Statistical significance is indicated as follows: * *p* < 0.05; ** *p* < 0.01; *** *p* < 0.001.

We collected 554 the CRGs by combining the “circadian rhythm” gene set from MSigDB (v2023.1) with findings from previously published studies. In the TCGA-LUAD cohort, differential expression analysis showed that some CRGs were clearly imbalanced in tumors compared to normal tissues. Many CRGs were either significantly upregulated or downregulated in tumor samples. These changes suggest that circadian rhythm pathways may be disrupted during the development and progression of lung adenocarcinoma. This pattern points to the possible role of circadian dysregulation in tumor biology and supports the need for deeper mechanistic research (Figure 1A). We carried out enrichment analysis on CRGs with differential expression. The findings revealed that these genes were mainly involved in several biological processes. These included circadian rhythm control, circadian-related behaviors, neurotransmitter receptor function, and pathways linked to the cell cycle (Figure 1B). These results indicate that disruption of the circadian rhythm might support tumor development by interfering with key pathways related to metabolism and cell growth. To investigate the potential prognostic role of CRGs, we performed univariate Cox regression analysis on genes with differential expression. The results of this analysis revealed several genes closely linked to overall survival. These genes might be useful as biomarkers to help divide patients into risk groups. They could also provide new targets for treatment in lung adenocarcinoma (Figure 1C). We identified the chromosomal positions of these survival-related genes throughout the genome. The corresponding results are presented in Figure 1D. These genes were distributed across multiple chromosomes, suggesting that circadian disruption may have a broad genomic effect in lung adenocarcinoma. For model development and external validation, we used the ComBat algorithm to correct batch effects between the TCGA and GEO datasets. PCA after correction showed that samples were well mixed and did not form separate clusters. This confirmed that the batch effects were effectively removed (Figure 1E).

### 3.2. Machine Learning-Based CRGs Risk Score Enables Prognostic Stratification

**Figure 2:**
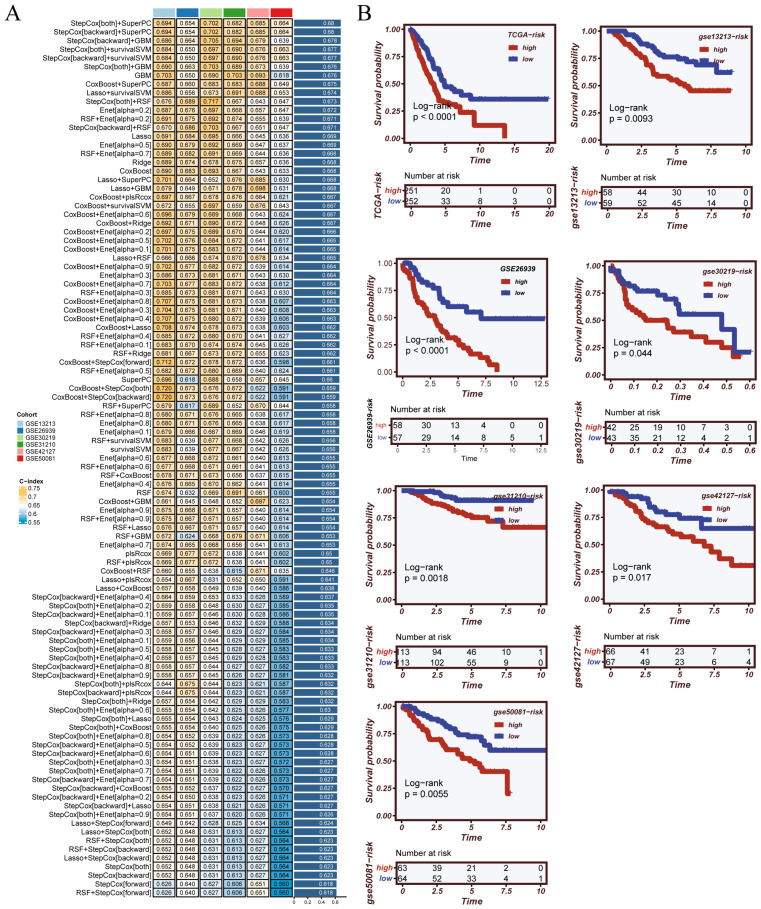
Evaluation and validation of the CRGs model. (**A**) C-index ranking of 101 machine learning algorithms derived from combinations of 10 machine learning algorithms across 6 training cohorts. The CRGs model constructed using StepCox[both] + SuperPC showed optimal and consistent performance. (**B**) Kaplan–Meier survival curves of patients stratified by the median CRGs risk score in the TCGA cohort and six external validation cohorts (GSE31210, GSE30219, GSE72094, GSE50081, GSE13213, GSE37745), indicating significant survival differences between high- and low-risk groups.

To develop a stable and widely applicable model for prognosis based on circadian rhythm–related genes, we used six transcriptome datasets from the GEO database. These datasets served as the training set for model construction. We created 101 machine learning algorithm combinations by integrating ten commonly used machine learning methods. Each model was trained and validated separately using strict 10-fold cross-validation within its dataset. We used the C-index as the main measure to evaluate model performance. Among all combinations tested, the model built with StepCox[both] and SuperPC consistently showed the best average C-index of 0.68 across the six training datasets. This high C-index indicates that the combination of StepCox[both] and SuperPC provided the most reliable and stable performance in prognostic prediction. Therefore, we selected this combination for the final model, as it demonstrated superior predictive accuracy and robustness compared to the other combinations tested. The results demonstrated that the model was stable and had good predictive performance (Figure 2A). So, we chose this combination as the final method for building the CRGs risk score model. Next, we applied the final CRGs model to the TCGA-LUAD dataset and six external validation datasets. In each dataset, patients were grouped into high-risk and low-risk categories according to the median CRGs score. Kaplan–Meier analysis revealed that, across all datasets, individuals in the high-risk group had significantly shorter overall survival (Figure 2B). These results confirmed that the CRGs model had strong and consistent prognostic value.

### 3.3. Robust Validation and Independent Prognostic Value of the CRGs Model Across Clinical and External Cohorts

**Figure 3:**
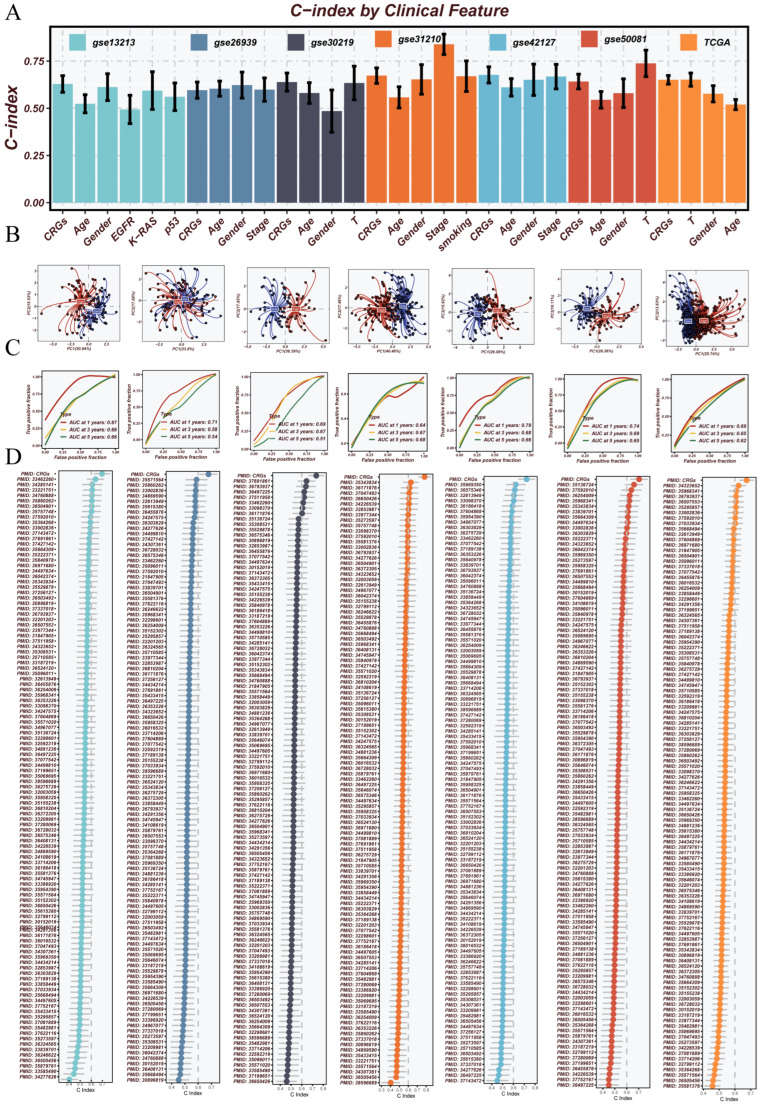
Multi-level performance evaluation of the CRGs model. (**A**) Comparison of the C-index of the CRGs model and common clinical features across seven datasets, demonstrating superior prognostic power of the CRGs classifier. (**B**) Principal component analysis (PCA) plots show clear separation between high- and low-risk groups, validating the model’s stratification capability. (**C**) ROC curves at 1-, 3-, and 5-year survival for each dataset, reflecting the model’s predictive accuracy across time points. (**D**) Benchmarking of the CRGs model against 113 alternative algorithmic combinations in 6 training cohorts, with CRGs ranking among the top-performing models.

To fully evaluate the performance and stability of the CRGs model across multiple datasets, we carried out a series of validation analyses. First, we compared the C-index of the CRGs risk score with several common clinical factors, including age, sex, tumor stage, and smoking history, across seven independent external cohorts (Figure 3A). The results showed that the CRGs model achieved higher C-index values than traditional clinical indicators in all datasets. This highlights its stronger ability for prognostic classification. To evaluate how well the model separated patients, we carried out principal component analysis (PCA). This method was used to observe the distribution of patients with different risk levels (Figure 3B). In all datasets, the PCA results showed a clear separation between the high-risk and low-risk groups. This suggests that the model has good power to distinguish prognostic subtypes. We then plotted ROC curves to evaluate survival prediction at 1, 3, and 5 years. This was achieved using data from the TCGA cohort and six independent validation datasets (Figure 3C). The CRGs model showed high AUC values in all datasets. This confirms the model’s strong and stable long-term predictive ability. Lastly, we compared the CRGs model with 114 published prognostic signatures reported in LUAD or other types of cancer (Figure 3D). These signatures were collected from peer-reviewed studies. The CRGs model consistently ranked among the top models based on C-index across datasets. This shows its broad applicability and excellent predictive performance.

**Figure 4:**
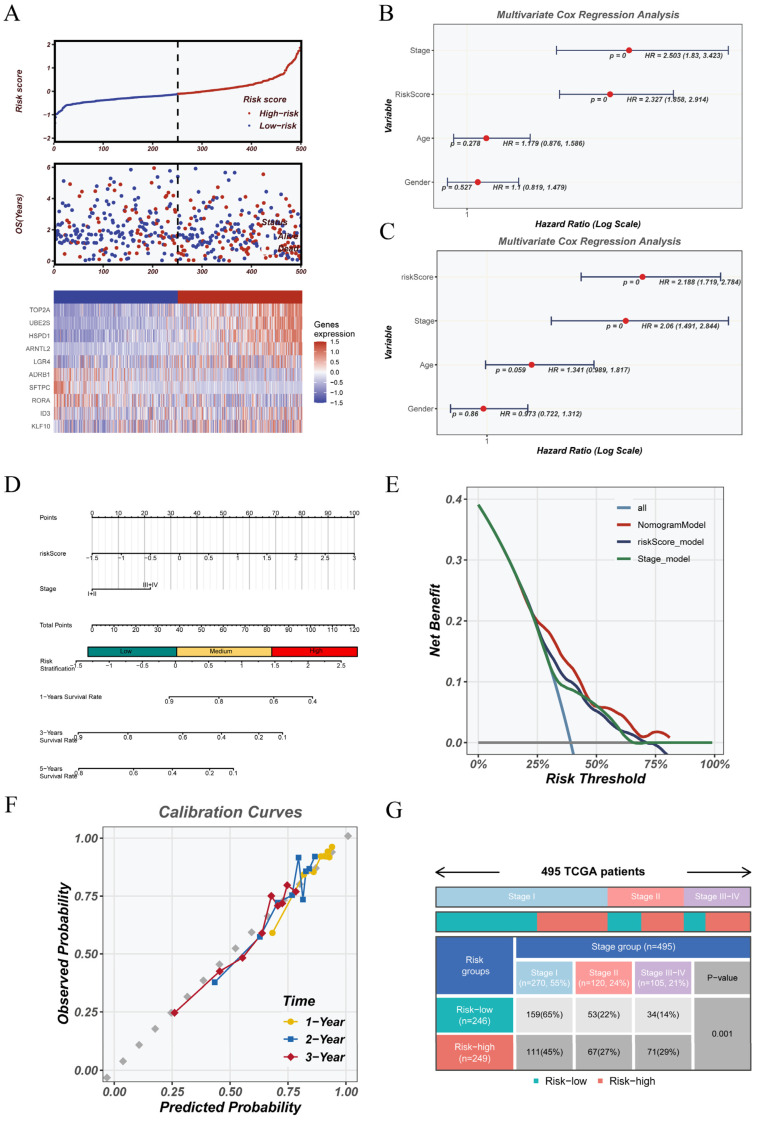
Comprehensive clinical evaluation of the CRGs model. (**A**) Risk score distribution, survival status, and expression heatmap of CRGs model genes in the TCGA cohort. Patients were classified into high- and low-risk groups based on the median risk score. (**B**) Univariate Cox regression analysis of clinical features and risk score. (**C**) Multivariate Cox regression analysis confirming the independent prognostic value of the CRGs score. (**D**) Nomogram integrating risk score and clinical variables (stage, age, gender) for individualized survival prediction. (**E**) Decision curve analysis (DCA) evaluating the net clinical benefit of the nomogram compared with models using only risk score or tumor stage. (**F**) Calibration plots for 1-, 2-, and 3-year survival probabilities, showing good concordance between predicted and observed outcomes. (**G**) Distribution of TNM stages across the high- and low-risk groups, demonstrating significant stage-based stratification.

To examine how the CRGs model relates to clinical outcomes in the TCGA cohort, we carried out several validation steps. Based on the median CRGs score, patients were grouped into high-risk and low-risk categories. Figure 4A displays the distribution of risk scores, overall survival status, and the expression heatmap of genes included in the model. The high-risk group showed a noticeably higher mortality rate than the low-risk group. Gene expression patterns also differed between the two groups, indicating distinct molecular profiles. To evaluate the predictive power of the CRGs risk score, we carried out univariate and multivariate Cox regression analyses. The models included clinical variables such as age, gender, and tumor stage. The analysis confirmed that the CRGs score remained an independent factor for predicting overall survival. This association stayed significant after adjusting for clinical variables (HR > 1, *p* < 0.05). The detailed results are shown in Figure 4B,C. Next, we built a nomogram that combined the CRGs score with clinical variables to predict 1-, 2-, and 3-year survival for each patient (Figure 4D). Decision curve analysis (Figure 4E) showed that this model gave better net clinical benefit than models using only stage or risk score, across a wide range of thresholds. The calibration plots in Figure 4F showed that the predicted survival rates matched the actual outcomes well at 1, 2, and 3 years. These findings supported the reliability and precision of the nomogram. We then compared the TNM stage distributions between the high- and low-risk groups (Figure 4G). Patients in the high-risk group were more often diagnosed at advanced stages. This difference reached statistical significance (*p* = 0.001). The findings indicate that a higher CRGs score may be associated with tumor development. This supports the clinical usefulness of the risk model.

### 3.4. Association Between CRGs Score and Immune Landscape

**Figure 5:**
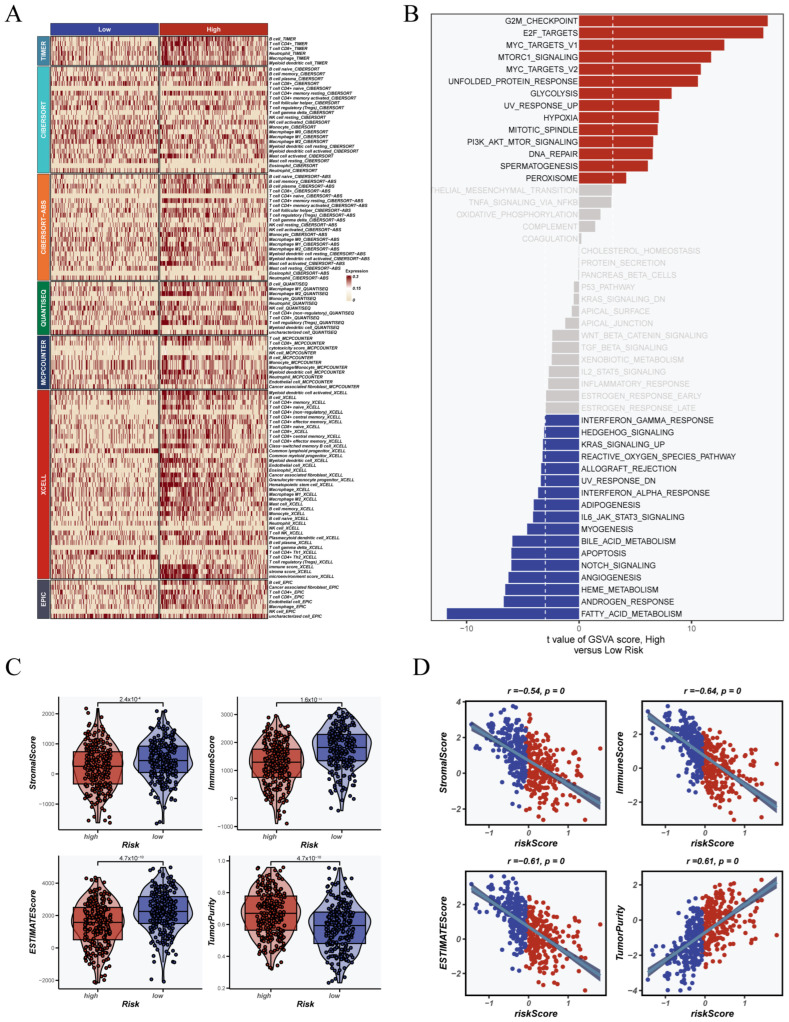
Immune landscape and functional differences between CRGs subgroups. (**A**) Heatmap showing immune infiltration levels estimated by seven different algorithms across low- and high-CRG groups. (**B**) GSVA enrichment analysis identifying differentially activated hallmark pathways between CRG groups. Red and blue bars represent pathways enriched in high- and low-CRG groups, respectively. (**C**) ESTIMATE algorithm evaluation of StromalScore, ImmuneScore, ESTIMATEScore, and tumor purity across CRG groups. (**D**) Correlation analysis between CRGs score and ESTIMATE-derived immune metrics.

To explore the immune relevance of the CRGs signature, we systematically analyzed the immune landscape and functional differences between CRGs-based groups. As presented in Figure 5A, seven established methods—CIBERSORT, TIMER, EPIC, XCELL, MCPCOUNTER, QUANTISEQ, and CIBERSORT-ABS—were used to estimate immune cell infiltration. The analysis revealed notable differences in the composition of immune cells between the high CRGs score group and the low CRGs score group. This suggests a possible link between the CRGs score and the immune background of the tumor. Gene set variation analysis (GSVA), shown in Figure 5B, revealed that patients with high CRGs scores had strong enrichment in tumor-promoting pathways. These included the G2M checkpoint, E2F target genes, and MYC-related pathways. Conversely, the low CRGs score group showed increased activity in pathways related to immunity and metabolism. These included interferon signaling, fatty acid processing, and T cell co-stimulation. These features suggest a more active immune microenvironment. We also used the ESTIMATE algorithm to evaluate stromal and immune components (Figure 5C). Patients in the high-CRGs group had lower StromalScore, ImmuneScore, and ESTIMATEScore. Their tumor purity was clearly higher. Correlation analysis (Figure 5D) confirmed these results. The CRGs score showed a strong negative correlation with stromal and immune scores (r < −0.6, *p* < 0.001) and a positive correlation with tumor purity (r > 0.6, *p* < 0.001). These findings support the idea that the CRGs model can stratify patients by immune infiltration and tumor immune status.

### 3.5. Multi-Algorithm Scoring Identifies Distinct Metabolic Profiles Across Single-Cell Types

**Figure 6:**
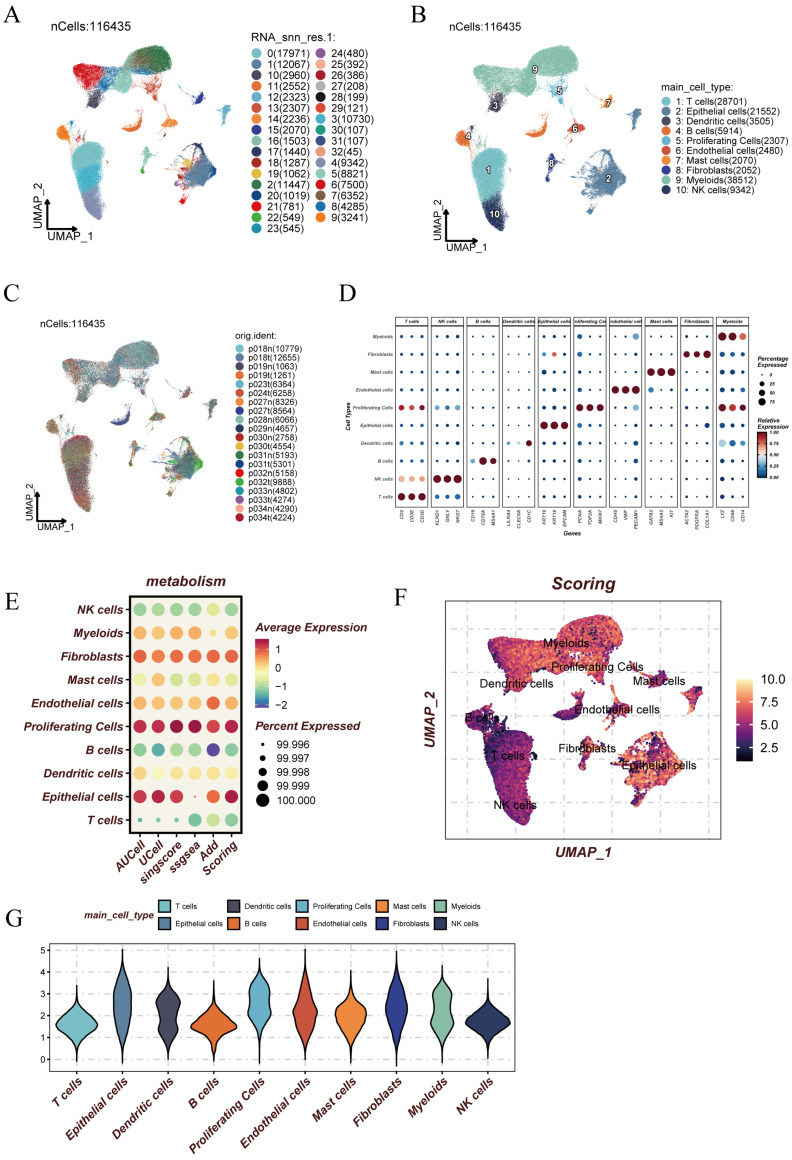
Multi-algorithm scoring identifies distinct metabolic profiles across single-cell types. (**A**) UMAP plot of 11,635 cells clustered into 33 distinct subpopulations. (**B**) Cell-type annotation showing 11 major immune and stromal lineages. (**C**) Cell distribution colored by patient of origin. (**D**) Dot plot showing representative marker gene expression used for cell-type identification. (**E**) Bubble plot summarizing metabolic activity scores across cell types based on five distinct algorithms. (**F**) UMAP visualization of global metabolic activity scores. (**G**) Violin plots comparing metabolic activity across cell types.

To better understand how the circadian rhythm gene (CRG) signature interacts with the tumor microenvironment, we built a single-cell transcriptomic map containing 11,635 cells from LUAD tissue samples. Before any downstream analysis, we applied a strict preprocessing pipeline. This included filtering out low-quality cells based on specific criteria. Cells with fewer than 500 detected genes, fewer than 1000 unique molecular identifiers (UMIs), or more than 20% of their transcripts derived from mitochondrial genes were excluded. These low-quality cells were removed to ensure the integrity of the data. These steps helped ensure the reliability of the results. Supplementary Figure S1 outlines the full procedure. As shown in Figure 6A, unsupervised clustering revealed 33 distinct transcriptional clusters. Using known marker genes (Figure 6B), we identified and labeled 11 major cell types, including immune and stromal populations such as T cells, epithelial cells, fibroblasts, and proliferating cells. Figure 6C shows how these clusters were distributed across different patients, showing good coverage and consistency. Expression of selected marker genes used for annotation is summarized in the dot plot (Figure 6D). To measure metabolic activity in each cell type, we used five scoring algorithms: AUCell, AddModuleScore, ssGSEA, SCSE, and Scoring. The results (Figure 6E) showed that non-immune cells—like epithelial cells, proliferating cells, and fibroblasts—had higher metabolic activity than immune cells like T cells and NK cells. The UMAP projection of global metabolic scores (Figure 6F) showed spatial heterogeneity in metabolic states across the tumor microenvironment. Violin plots in Figure 6G also confirmed significantly higher metabolic scores in non-immune cell types, suggesting active metabolic programs in malignant and stromal regions.

### 3.6. Metabolic Activity and Cell–Cell Communication in CRGs-Based Subtypes

**Figure 7:**
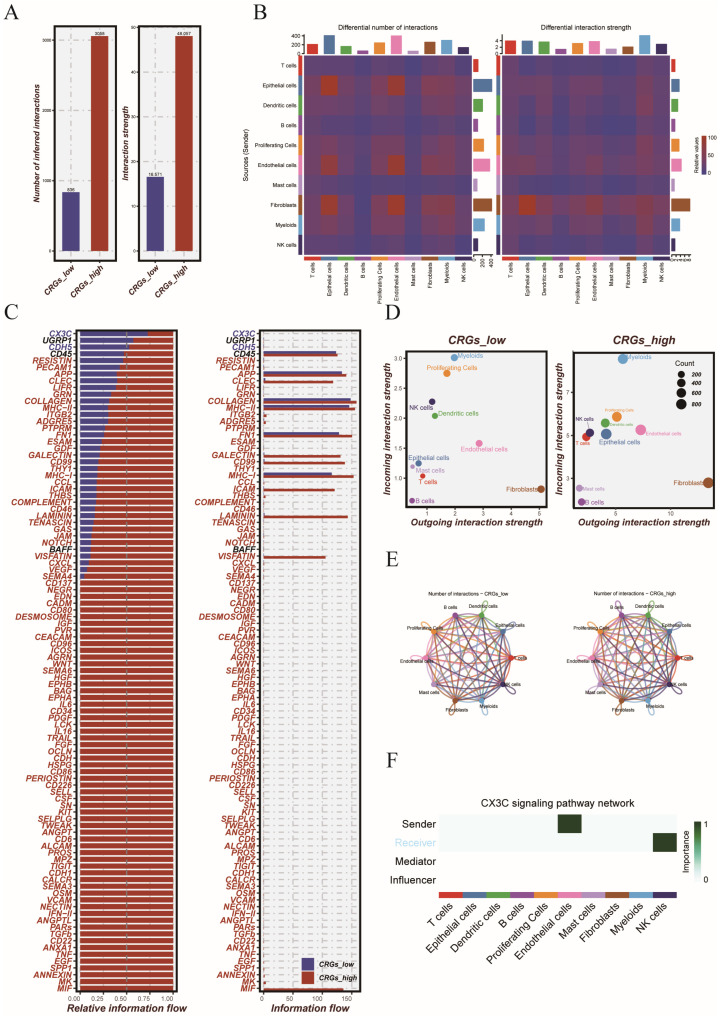
Cell–cell communication analysis between CRGs_low and CRGs_high groups. (**A**) Bar plots comparing the overall number (**left**) and strength (**right**) of inferred cell–cell interactions between CRGs_low and CRGs_high groups. (**B**) Heatmaps displaying the differential interaction number (**left**) and interaction strength (**right**) among major cell types. (**C**) **Left**: Relative information flow of signaling pathways in CRGs_high versus CRGs_low groups; **Right**: absolute information flow comparison for each pathway between the two groups. (**D**) Scatter plots showing outgoing and incoming interaction strength of each cell type, highlighting communication roles within the CRGs_low and CRGs_high groups. (**E**) Circle plots visualizing global communication patterns across cell types in CRGs_low and CRGs_high states. (**F**) Role annotation within the CX3C signaling pathway, including sender, receiver, mediator, and influencer cell types.

To explore how CRGs-based metabolic subtypes affect cell–cell communication, we used CellChat analysis to compare signaling patterns between the two CRGs-defined risk categories Figure 7A shows that the CRGs_high group exhibited a greater number of cell–cell interactions. The strength of these interactions was also notably higher compared to the other group. This suggests that metabolically high-risk tumors have more complex and active signaling networks. The heatmap results showed that epithelial cells, endothelial cells, and fibroblasts were the main contributors to the differences in interaction patterns (Figure 7B). At the pathway level, we found that the CX3C signaling pathway was mainly enriched in the CRGs_low group. Conversely, the CRGs_high group showed strong activation of the MIF, SPP1, and CDH signaling pathways (Figure 7C). This suggests distinct molecular communication features under different metabolic states. At the cell type level, signaling role analysis showed that fibroblasts were the major signal senders in both groups, while myeloid cells were the main signal receivers (Figure 7D). Overall, the CRGs_high group showed a more interconnected communication network (Figure 7E). Focusing on the CX3C pathway, which was specifically active in the CRGs_low group, we found that dendritic cells were the main source of signals. Fibroblasts acted as both mediators and receivers (Figure 7F). This suggests the presence of a unique immune-regulatory axis in metabolically low-risk tumors.

### 3.7. Genomic Instability Landscape Associated with CRGs Signature

**Figure 8:**
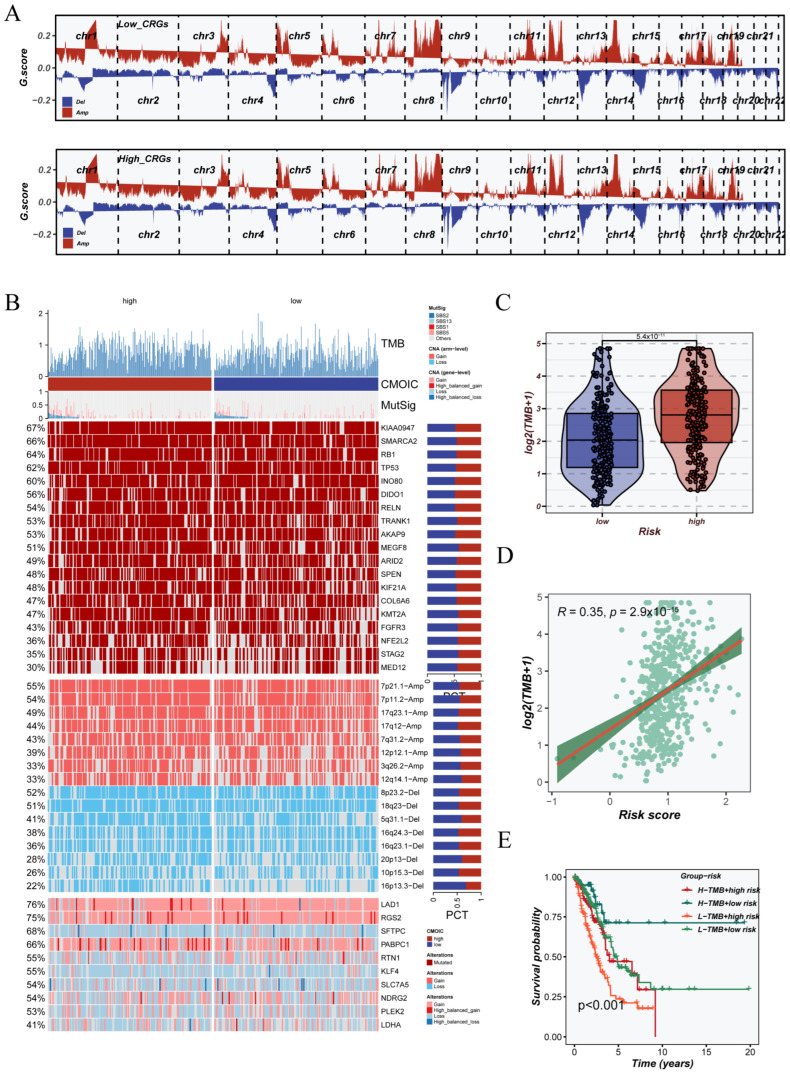
Genomic alteration landscape and tumor mutational burden (TMB) across CRGs subtypes. (**A**) Copy number variation (CNV) profiles inferred using GISTIC2.0 algorithm for low_CRGs and high_CRG groups, where red and blue segments represent amplification and deletion events, respectively. (**B**) Oncoprint showing top mutated genes (mutation frequency > 5%) and representative mutational signatures stratified by CRGs subtypes. (**C**) Comparison of log2-transformed TMB levels between high and low CRG groups. (**D**) Spearman correlation analysis between CRGs risk score and TMB. (**E**) Survival analysis integrating CRGs score and TMB status; patients were divided into four combined groups using optimal TMB cutoff.

To explore the link between CRGs subtypes and genomic instability, we analyzed somatic copy number variations (CNVs) and mutation profiles in the TCGA-LUAD cohort. We used the GISTIC2.0 tool to estimate CNVs. The results showed clear differences in amplification and deletion patterns between the CRGs subgroups. As shown in Figure 8A, the high_CRGs group had widespread amplifications on chromosomes 3, 5, and 7, and frequent deletions on chr8p and chr9p. In contrast, the low_CRGs group showed a more stable chromosomal structure. The mutation landscape of LUAD patients (Figure 8B) revealed that several genes, including TP53, TTN, and KRAS, were frequently mutated in both groups. Although mutation frequency tended to be elevated in the CRGs-high subgroup, we further assessed tumor mutational burden (TMB) and observed a marked increase in TMB values compared to the CRGs-low group (*p* < 0.001; Figure 8C). Correlation analysis showed a moderate positive association between CRGs risk scores and TMB (Spearman R = 0.35, *p* = 2.9 × 10^−15^; Figure 8D). Additional survival analysis revealed that individuals with both elevated CRGs scores and high TMB had the poorest prognosis (Figure 8E). These results indicate that integrating CRGs-based risk with TMB enhances prognostic prediction.

### 3.8. Immunological Status Assessment and Immunotherapy Prediction Based on CRG Stratification

**Figure 9:**
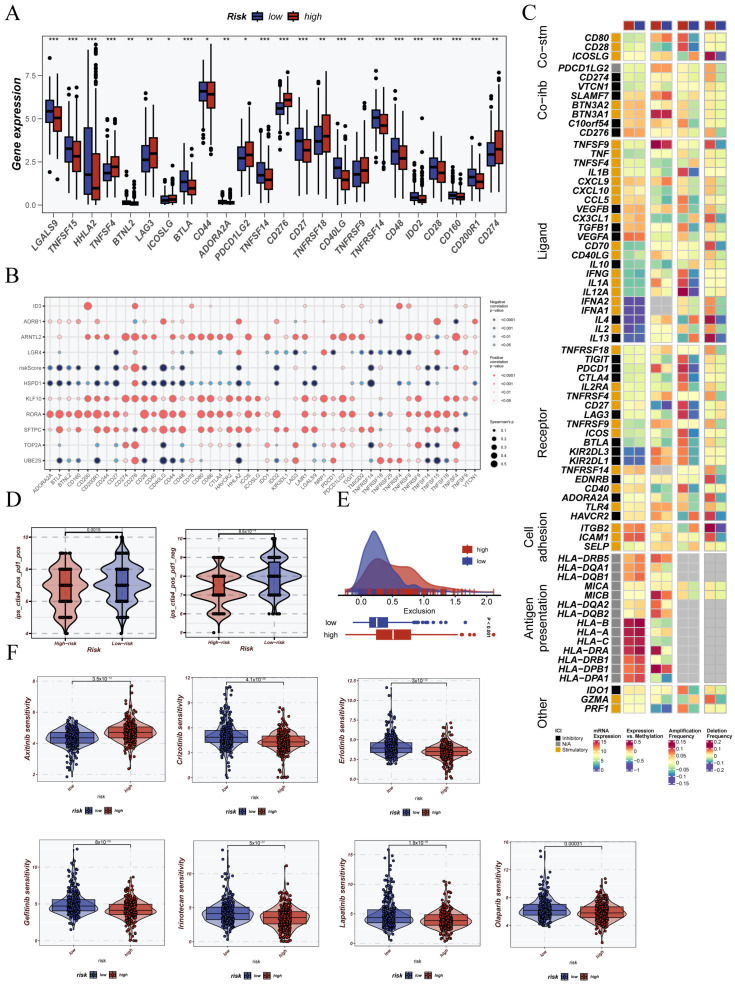
Comprehensive assessment of immune response and therapeutic implications based on CRGs signature. (**A**) Differential expression of immune checkpoint-related genes between high and low CRG groups. (**B**) Spearman correlation between immune checkpoint genes and CRGs score. (**C**) Heatmap of immune regulatory factors including co-stimulatory, co-inhibitory molecules, ligands, receptors, antigen presentation genes, and cell adhesion markers. (**D**) Immunophenoscore (IPS) comparison from the TCIA database between CRGs subgroups. (**E**) Exclusion score from the TIDE algorithm indicating immune evasion potential. (**F**) Sensitivity analysis of chemotherapeutic agents between CRGs subgroups. Statistical significance is indicated as follows: * *p* < 0.05; ** *p* < 0.01; *** *p* < 0.001.

In addition, the expression of most immune checkpoint markers was significantly increased in patients classified as high risk. This finding indicates that these patients may have a tumor microenvironment with stronger immunosuppressive features (Figure 9A). Next, we performed Spearman correlation analysis. The analysis revealed a strong positive association between the CRGs score and immune checkpoint gene expression. This supports a possible link between circadian rhythm regulation and immune escape (Figure 9B). We also examined the expression of several immune regulatory factors, including immunostimulatory and immunosuppressive genes, chemokines, and their receptors. A large portion of these genes showed higher expression in the high CRGs score group. This further supports the idea that CRGs characteristics are strongly linked to immune regulation (Figure 9C). According to the TCIA database, patients with low CRGs scores had significantly elevated IPS values. This indicates a more favorable immune response profile in this group (Figure 9D). Conversely, patients in the high CRGs score group had notably higher exclusion scores based on the TIDE analysis. This finding supports the presence of an immune-excluded phenotype in this group (Figure 9E). Lastly, drug sensitivity prediction using the GDSC database showed that drugs like apatinib and gefitinib were more effective in patients with low CRGs scores. This suggests that the CRG signature may also help guide treatment choices (Figure 9F).

### 3.9. Pan-Cancer Prognostic Significance and Functional Impact of ARNTL2 in LUAD

**Figure 10:**
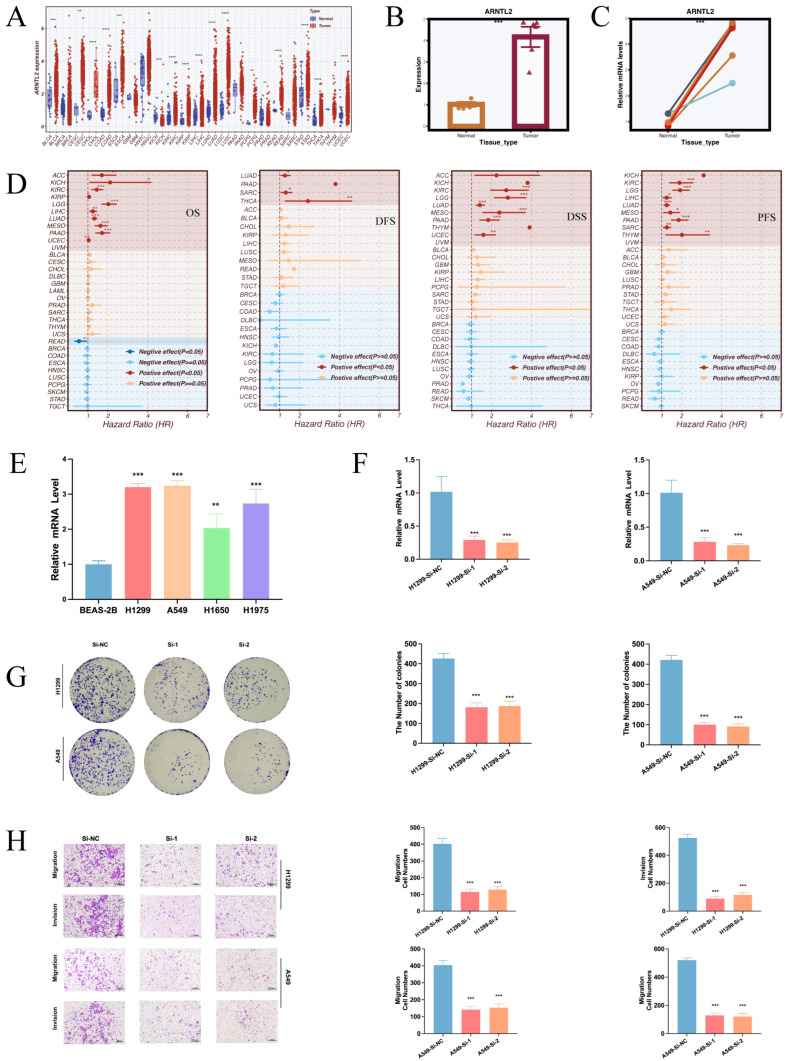
Functional validation of the model gene ARNTL2. (**A**) Pan-cancer expression profile of ARNTL2 showing elevated expression in multiple tumors. (**B**,**C**) qRT-PCR analysis of ARNTL2 mRNA levels in LUAD tumor versus adjacent tissues from Tianjin Chest Hospital. (**D**) Univariate Cox regression analyses across OS, DFS, DSS, and PFS outcomes indicating ARNTL2 as a prognostic factor in various cancers. (**E**) ARNTL2 expression in LUAD cell lines compared to normal lung epithelial cells (BEAS-2B). (**F**) Validation of ARNTL2 knockdown efficiency in A549 and H1299 using siRNA. (**G**) Colony formation assays demonstrating impaired clonogenic ability upon ARNTL2 knockdown. (**H**) Transwell migration and invasion assays showing reduced cell motility following ARNTL2 silencing. Statistical significance is indicated as follows: * *p* < 0.05; ** *p* < 0.01; *** *p* < 0.001; **** *p* < 0.0001.

After building the CRGs risk signature, we aimed to select a key representative gene for further functional studies. ARNTL2 is a core regulator in the circadian rhythm system. It plays an important role in maintaining cellular homeostasis, responding to stress, and regulating metabolism. In our analysis, ARNTL2 was strongly enriched in circadian-related pathways and showed a high contribution in the CRGs scoring system. Since few studies have focused on ARNTL2 in LUAD, we chose this gene for further validation. First, we examined ARNTL2 expression across multiple cancer types. We found that ARNTL2 was broadly upregulated in many solid tumors (Figure 10A). Surgical specimens collected from Tianjin Chest Hospital were analyzed, and the results of qRT-PCR showed that ARNTL2 mRNA expression was significantly elevated in LUAD tumor tissues compared to adjacent normal tissues (Figure 10B,C). Univariate Cox analysis across multiple cancer types revealed that elevated ARNTL2 expression was consistently associated with worse outcomes in OS, DFS, DSS, and PFS (Figure 10D). At the cell line level, ARNTL2 showed high expression in several lung cancer models, especially in H1299 and A549 cells (Figure 10E). We selected these two cell lines for functional experiments and confirmed ARNTL2 knockdown by qRT-PCR (Figure 10F). Functional assays showed that ARNTL2 knockdown clearly reduced colony formation ability (Figure 10G). Transwell migration and invasion assays also revealed that cells had weaker migratory and invasive abilities after ARNTL2 was silenced (Figure 10H). Overall, the findings indicate that ARNTL2 may function as an oncogenic factor in LUAD. It might also play an important role in the CRGs-based risk model.

## 4. Discussion

LUAD, the most prevalent subtype of non-small cell lung cancer (NSCLC), remains a leading cause of cancer-related mortality worldwide [1]. It shows strong intratumoral heterogeneity. It also often develops resistance to treatment. In addition, there is a lack of reliable markers to predict response to immunotherapy. These issues make clinical management very challenging. Some patients with LUAD have responded well to immune checkpoint inhibitor (ICI) therapy, but only a small portion of patients respond well. This highlights the need to find stable molecular signatures to help predict outcomes and guide treatment choices. Based on this background, we conducted a systematic investigation into the function of the CRGs in LUAD. We also built a new CRGs-based risk model. This model aims to reveal the prognostic value of CRGs, their links to immune phenotypes, and their possible biological functions.

Circadian rhythm controls many basic physiological processes. These include cell cycle regulation, metabolism, DNA repair, and immune homeostasis. When circadian control is disrupted, it may lead to tumor development and cancer progression in several malignancies [42]. However, the overall landscape and biological roles of the CRGs in LUAD are still not well understood. In this study, we collected a panel of 554 CRGs. We used a strict machine learning workflow to build a risk model. We tested 101 algorithm combinations and selected StepCox[both] combined with SuperPC based on C-index ranking. The final CRGs score showed strong and stable prognostic performance in multiple independent LUAD cohorts. It also outperformed previously published prognostic models. Compared to traditional LASSO-Cox methods, our multi-algorithm strategy reduced overfitting. It improved generalizability and produced a risk tool with practical clinical value.

To further explore the immune relevance of CRGs stratification, we used scRNA-seq data to analyze the tumor immune microenvironment. In the low-CRGs group, we observed stronger activity of the CX3C signaling axis, especially the CX3CL1–CX3CR1 interaction between mast cells and NK cells. This chemokine axis is known to help recruit and activate cytotoxic immune cells. It creates a pro-inflammatory and immune-active environment that may help suppress tumor growth. Conversely, tumors with high CRGs scores showed notable activation of several immunosuppressive pathways, such as MIF, SPP1, and CDH. Among them, MIF plays a key role in regulating myeloid-derived suppressor cells (MDSCs) and tumor-associated macrophages (TAMs). It can promote T cell exhaustion and immune escape. SPP1 supports extracellular matrix remodeling and TAM recruitment. CDH molecules, like CDH1, may reduce antigen presentation and promote immune exclusion. These findings suggest that CRGs stratification reflects distinct immune environments. Tumors with low expression levels of CRGs show signs of stronger immune surveillance, while tumors with high expression of CRGs tend to show features of immune escape.

In addition, we identified ARNTL2 as a key gene in the CRGs’ signatures and carried out comprehensive functional validation. ARNTL2 is a core transcriptional regulator of the circadian rhythm. Previous studies have shown that it can promote tumor progression by triggering the PI3K/AKT and MAPK signaling pathways. It also contributes to metabolic changes and facilitates epithelial–mesenchymal transition (EMT). Our analysis showed that ARNTL2 was highly expressed in LUAD tissues and cell lines. Its high expression was strongly associated with poor prognosis, increased TMB, and elevated TIDE Exclusion scores. These findings suggest a possible role in resistance to immunotherapy. Functional experiments confirmed that knocking down ARNTL2 reduced LUAD cell proliferation, migration, and colony-forming ability. This further supports its oncogenic role. These results indicate that ARNTL2 may serve as a promising biomarker and a potential therapeutic target in LUAD. It may act through a circadian–metabolic–immune regulatory axis.

## 5. Conclusions

Taken together, we constructed and validated a prognostic model derived from CRGs. This model shows strong potential for clinical application. We combined bulk and single-cell transcriptomic data to create a robust prediction tool. At the same time, we uncovered immune-related mechanisms and identified key pathways and regulators that shape the tumor microenvironment. However, this study has some limitations. Firstly, our results are mainly based on retrospective datasets. Future work should include prospective validation in larger, multi-center clinical cohorts. Secondly, although we used a diverse modeling strategy, the real-world performance across different populations and treatment settings still needs further testing. Thirdly, while we gained some early functional insights, in vivo experiments are still needed to confirm the proposed mechanisms, especially those involving ARNTL2 and the CX3C axis.

Looking ahead, future studies can explore whether targeting circadian genes can improve responses to immunotherapy. The use of spatial transcriptomics and proteomics may also help reveal how CRGs drive immune heterogeneity in tumors. Overall, our findings provide a new framework for CRG-based immune classification and personalized treatment, laying a foundation for future progress in LUAD and circadian-oncology research.

## Data Availability

The scRNA-seq data used in this study were obtained from a previously published and publicly accessible dataset (PMID: 34663877). Details regarding the specific repositories and accession numbers are provided in the main text and Supplementary Materials.

## Supplementary Materials

The following supporting information can be downloaded at: https://www.mdpi.com/article/10.3390/cancers17172911/s1, Figure S1: The preprocessing pipeline of single-cell transcriptomic map. Table S1: Data sets resources included in this study. Data sets resources included in this study. Table S2: Primers and siRNA Sequences Used in the Study.

## Abbreviations

The following abbreviations are used in this manuscript:

LUAD	lung adenocarcinoma
GSVA	gene set variation analysis
TCGA	The Cancer Genome Atlas
qRT-PCR	quantitative real-time PCR
TME	tumor microenvironment
IPS	immunophenoscore
TCIA	The Cancer Immunome Atlas
TIDE	tumor immune dysfunction and exclusion
