# Supplementary figures and images for: Application of Single-Cell Sequencing and Machine Learning in Prognosis and Immune Profiling of Lung Adenocarcinoma: Exploring Disease Mechanisms and Treatment Strategies Based on Circadian Rhythm Gene Signatures
Source: Cancers (Basel). 2025 Sep 5;17(17):2911. doi: 10.3390/cancers17172911 (PMC12428086; doi:10.3390/cancers17172911)

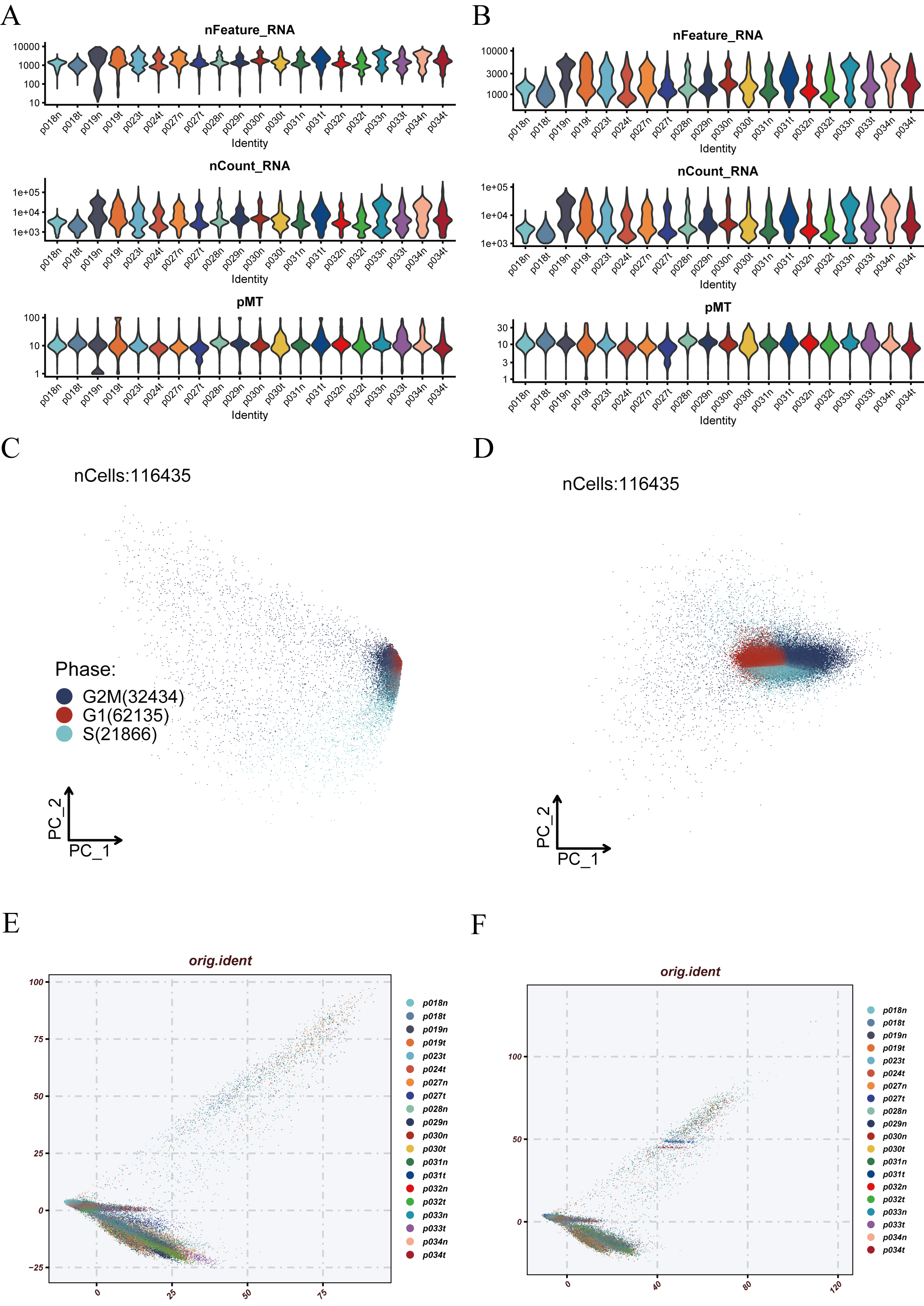

Supplement: Supplementary file 1 [file cancers-17-02911-s001.zip › supplementary/Supplement Figure S1.tif]
